# A neural network based global traveltime function (GlobeNN)

**DOI:** 10.1038/s41598-023-33203-1

**Published:** 2023-05-03

**Authors:** Mohammad H. Taufik, Umair bin Waheed, Tariq A. Alkhalifah

**Affiliations:** 1 Physical Science and Engineering Division, King Abdullah University of Science and Technology, 23955 Thuwal, Saudi Arabia; 2 Department of Geosciences, King Fahd University of Petroleum and Minerals, 31261 Dhahran, Saudi Arabia

**Keywords:** Geophysics, Computational science

## Abstract

Global traveltime modeling is an essential component of modern seismological studies with a whole gamut of applications ranging from earthquake source localization to seismic velocity inversion. Emerging acquisition technologies like distributed acoustic sensing (DAS) promise a new era of seismological discovery by allowing a high-density of seismic observations. Conventional traveltime computation algorithms are unable to handle virtually millions of receivers made available by DAS arrays. Therefore, we develop GlobeNN—a neural network based traveltime function that can provide seismic traveltimes obtained from the cached realistic 3-D Earth model. We train a neural network to estimate the traveltime between any two points in the global mantle Earth model by imposing the validity of the eikonal equation through the loss function. The traveltime gradients in the loss function are computed efficiently using automatic differentiation, while the P-wave velocity is obtained from the vertically polarized P-wave velocity of the GLAD-M25 model. The network is trained using a random selection of source and receiver pairs from within the computational domain. Once trained, the neural network produces traveltimes rapidly at the global scale through a single evaluation of the network. As a byproduct of the training process, we obtain a neural network that learns the underlying velocity model and, therefore, can be used as an efficient storage mechanism for the huge 3-D Earth velocity model. These exciting features make our proposed neural network based global traveltime computation method an indispensable tool for the next generation of seismological advances.

## Introduction

Traveltime modeling is an essential component of modern seismological studies with applications in earthquake source localization^1–4^, earthquake early warning systems^5,6^, seismic velocity inversion^7–12^, and earthquake source parameter estimation^13^. Recent advances in seismological instrumentation have seen the emergence of fiber-optic Distributed Acoustic Sensing (DAS) technology as a dense array of strain sensors for continuous and real-time seismic monitoring^14^. Conventional finite-difference-based traveltime algorithms are computationally intractable for handling millions of virtual receivers provided by DAS arrays for large 3-D surveys, like the US seismological array. Although a standard first-order conventional eikonal solver is mostly employed in practice^15^, their efficiency and accuracy limits hamper its practical application for inverse problems at such scales, and especially for real time applications. Therefore, to extract full value from dense 3-D seismic data sets, an alternative approach is needed that could model seismic traveltimes efficiently between any two points. One way to obtain this efficiency can be achieved through forming a functional that inherently stores the traveltime between two points, and as result has the velocity model information embedded.

Currently, seismic traveltimes are computed by numerically solving the eikonal equation, which is a first-order nonlinear partial differential equation (PDE) and can be derived from both the wave equation via the Wentzel-Kramers-Brillouin approximation or Huygens’ principle using ray theory^16^. It is essentially used to address two fundamental questions pertaining to traveling seismic waves: (i) What paths do these waves take in traveling between any two points of interest? (ii) How long do they take in doing so? Seismologists use this information for locating earthquakes and performing subsequent downstream seismological analyses, including seismic tomography and earthquake property estimation.

Throughout the past decades, geometric ray theory has been an established field to solve the seismic tomography problem. Being an important part during the forward problem (modeling traveltimes), methods based on this approach can be categorized into two major groups; ray-based and grid-based approaches. The former^17–20^, relies on solving the characteristic equation derived from the high-frequency asymptotic assumption of the wave equation, the eikonal, while the later directly solves the eikonal equation. The ray-based (e.g., two-points ray-tracing^21^) approach has the advantage of being able to track multi-arrivals, as compared to the grid-based eikonal solvers, which primarily tracks the first-arrivals. For a strongly heterogeneous medium, however, ray-based methods often fail to solve for the traveltime as rays may diverge, and thus eikonal solvers is a more suitable solution^22^. Nevertheless, the governing PDE for traveltimes under the high frequency asymptotic approximation of the wave equation is the eikonal equation. Thus, both numerical ray-based methods and direct finite-difference methods have been utilized in traveltime tomography^23–27^.

Several finite-difference-based algorithms have been deployed over the years to solve the eikonal equation^28,29^. However, these methods suffer from a number of limitations. Primarily, they are limited by computational bottlenecks when repeated traveltime computations are needed for perturbations in the earthquake source location or the seismic velocity model. Moreover, in the case of dense seismic networks, the finite-difference grid has to be chosen accordingly, requiring prohibitively large disk storage, causing a further strain on the computational resources. Advances in the field of scientific machine learning offer new pathways to address these outstanding challenges and usher in a new era of scientific discovery in Earth sciences.

Physics-informed machine learning^30,31^ has been very useful in addressing various problems in computational sciences^32–35^. In seismology, they have demonstrated efficacy on both forward and inverse problems at local and regional scales based on wave fields^36,37^ and traveltimes^38–41^. Such physics-informed neural networks (PINNs) leverage the capabilities of deep neural networks as universal function approximators^42^. Contrary to purely data-driven deep learning approaches, PINNs restrict the space of admissible solutions by enforcing the validity of the underlying partial differential equation governing the actual physics of the problem. This is achieved by using automatic differentiation^43^ to compute gradients of the neural network’s output with respect to its inputs.

We harness the capabilities of neural networks as function approximators to learn a traveltime map for the global mantle Earth model. By minimizing a loss function formed by imposing the validity of the underlying eikonal equation, a neural network is trained to produce traveltime solutions between any points in a 3-D Earth model. Specifically, we use automatic differentiation to compute the spatial gradients of traveltime fields, which are then used to obtain a recovered velocity model using the eikonal equation. Then, the neural network training process aims at minimizing the difference between the predicted and the provided target velocity models. For the target velocity model, we use the GLAD-M25 model^44^. The term *global* used throughout the article refers to the *global* mantle velocity model used as the input.

Our proposed framework allows the neural network to learn a traveltime function that is mesh-free and can be used to instantly evaluate the traveltime between any two points in the 3-D Earth model. This allows us to avoid storing traveltime lookup tables, as the traveltimes can be generated on the fly using the trained neural network. This ensures that the method scales independently of the number of seismic stations and has a compact memory footprint. Moreover, the obtained traveltime solution is guaranteed to be differentiable with respect to the source or receiver locations. This allows our trained neural network to be used for a variety of seismological applications at the global scale. These exciting features offer a promising alternative in seismic traveltime modeling at the global Earth scale, and our trained PINN model can be used as an efficient modeling engine for seismological inverse problems. As a byproduct of the training process, we obtain a neural network that learns the underlying velocity model and, therefore, it can also be used as an efficient storage mechanism for the huge 3-D Earth velocity model, which can be queried for further applications, avoiding the I/O bottleneck.

**Figure 1:**
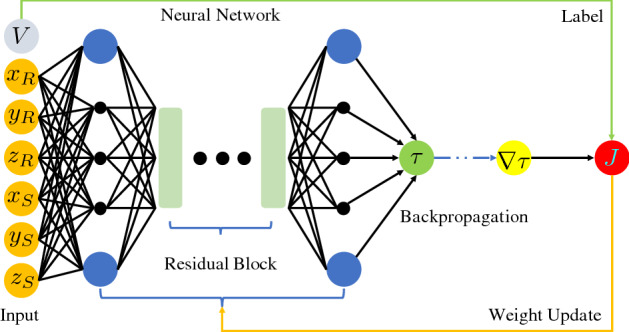
Physics-informed neural network based global seismic traveltime modeling. A neural network consisting of 20 residual blocks along with a fully-connected layer on either sides of it is used. Each of these layers contains 512 neurons. The input to the network are the source and receiver coordinates, while the output is the traveltime factor $\tau$. The spatial gradients of the traveltime factor $\nabla \tau$ are obtained through automatic differentiation. These gradients along with the input velocity information are used to form the training loss function *J*. Once the network is trained, it can be used to instantly evaluate traveltimes between any two points in the global Earth model.

## Results

To demonstrate the ability of our neural network function (Fig. 1) in rapid modeling of global seismic traveltimes, we perform several numerical tests to analyze its accuracy, robustness, and generalization ability. We group these tests into two main categories. In the first case, we consider a single earthquake located inside the Earth and train the PINN model for evaluating traveltimes from this point-source to any location on the surface of the Earth or the interior of it down to the outer core boundary. Next, we consider a more realistic case using 2234 seismic stations from the USArray, which covers the entire contiguous United States and parts of Canada. We analyze the performance of GlobeNN in rapidly computing traveltimes from any candidate point-source inside the Earth to the USArray stations. We also evaluate the extrapolation ability of our trained model in predicting traveltimes to stations outside the USArray domain. To analyze the performance of GlobeNN for these tests, we compare the target GLAD-M25 velocity model with the one recovered using the traveltimes predicted by the trained PINN model and computed using Eq. (5).

### Point-source traveltime modeling

**Figure 2:**
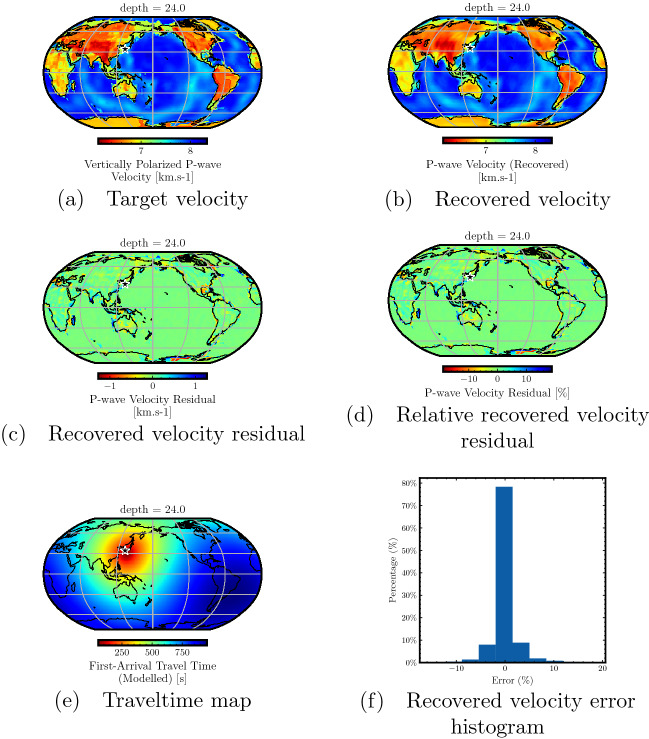
Accuracy analysis for the traveltimes predicted by the neural network trained for a point-source using model depth of 24 km. The target P-wave velocity model from the GLAD-M25 model (**a**) and the one recovered using the predicted traveltime map (**b**) at a depth of 24 km show similar macro trends, highlighting the accuracy of the traveltime predictions. The velocity residual (**c**) and the relative velocity residual (**d**) show that for the most part, the difference is close to zero. Some differences are observed in the boundary zone between the oceanic and continental lithosphere due to sharp velocity variations that are not fully captured by the trained PINN model. The traveltime map from the considered point-source (**e**) highlights that the method can be used for global seismic traveltime computation on a dense grid of stations. The recovered velocity error histogram (**f**) shows that for a majority of the geographical region at this depth, the errors are close to zero, highlighting the accuracy of PINN-based traveltime prediction despite the complexities of the Earth’s lithosphere.

**Figure 3:**
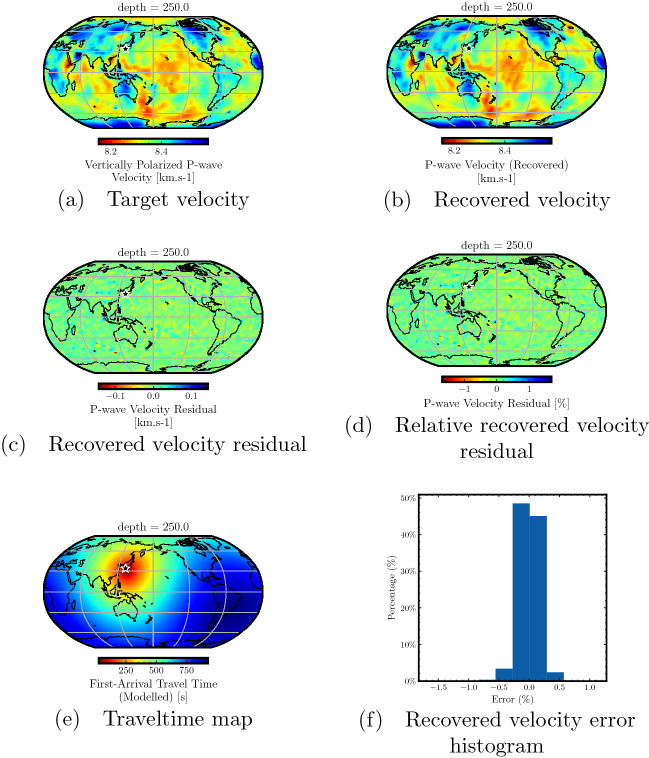
Accuracy analysis for the traveltimes predicted by the neural network trained for a point-source using model depth of 250 km. The target P-wave velocity model from the GLAD-M25 model (**a**) and the one recovered using the predicted traveltime map (**b**) at a depth of 250 km show striking similarity. The velocity residual (**c**) and the relative velocity residual (**d**) show that the difference is close to zero throughout the model at this model depth. The relatively smoother velocity variations at this depth compared to the lithosphere are well captured during the neural network training. The traveltime map from the considered point-source (**e**) highlights that the method can be used for global seismic traveltime computation on a dense grid of stations. The recovered velocity error histogram (**f**) shows that for the most part, the errors are between − 0.5 and 0.5%, highlighting the accuracy of PINN-based traveltime prediction.

In this case, we examine the 2001 south of Honshu, Japan earthquake (Mw 6.8, mb 6.4, Ms 6.5). It was situated at 33.97$^{\circ }$ N, $132.52^{\circ }$ E, and had a depth of 47.4 km, and shown with a black star in Figs. 2, 3. We consider a random selection of receiver points spread throughout the GLAD-M25 velocity model and train our neural network to minimize the loss function given in Eq. (7). Once the training is completed, we evaluate traveltimes to all points of the discretized GLAD-M25 model emanating from the considered point-source. This traveltime map is then used to compute the corresponding (recovered) P-wave velocity through Eq. (5). To analyze the performance of the traveltime predictions, we compare the recovered velocity obtained from the predicted traveltimes with the reference GLAD-M25 velocity model at depths of 24 km and 250 km. These depths are chosen to demonstrate the varying accuracy of traveltime predictions for regions with different velocity structures.

In Fig. 2, we analyze the performance of traveltime prediction represented by the recovered velocity at a depth of 24 km. This allows us to demonstrate the challenge associated with accurate traveltime computation for the highly heterogeneous lithosphere. Figure 2a shows the target velocity from the GLAD-M25 model, whereas Fig. 2b shows the recovered velocity, both at a depth of 24 km. We observe close similarity in the macro-trends of the two velocities. To analyze the differences, we plot the residual between the target and the recovered velocities in Fig. 2c and the relative residual in Fig. 2d. We observe that the recovered velocity is accurate for most of the geographical area at this depth, although some errors are noticeable mainly at the boundary between the oceanic and continental lithosphere, where we have sharp variations in the velocity model. This is understandable due to the spectral bias of neural networks as they favor learning a smoother representation of the underlying function and take considerably longer training times to approximate high-frequency features in the underlying solution^45^. Despite the complex nature of Earth’s lithosphere, the recovered P-wave velocity indicates that the PINN model is able to compute traveltimes with high accuracy. Figure 2e shows the traveltime map from the considered point-source to all points at a depth of 24 km. The trained neural network provides a traveltime function defined over a continuous domain. Thus, it can be evaluated at any arbitrary point within the computational domain. Finally, in Fig. 2f, we plot the residual histogram for all points at a depth of 24 km, confirming that the error is close to zero for the majority of the area at this depth.

In Fig. 3, we analyze the performance of traveltime prediction considering recovered velocity in the upper mantle region at a depth of 250 km. Figure 3a,b show the target and recovered velocities at this depth, respectively, indicating close similarity between the two. Figure 3c plots the residual between the recovered and target velocities and Fig. 3d shows the relative residual. We observe negligible errors at this depth throughout the entire geographical area, indicating high accuracy of traveltime prediction. Compared with the prior analyzed depth of 24 km, we observe even improved accuracy as the target velocity at this depth does not contain sharp velocity variations as in the complex lithosphere. This allows the PINN model to learn the underlying function accurately, which translates into the accuracy of the traveltime predictions. In Fig. 3e, we plot the traveltime map from the considered point-source to all points at a depth of 250 km. The recovered velocity histogram at this depth is plotted in Fig. 3f showing that the majority of the relative residual lies between -0.5 to 0.5%, indicating an accuracy of 99% for most of the computational domain.

**Figure 4:**
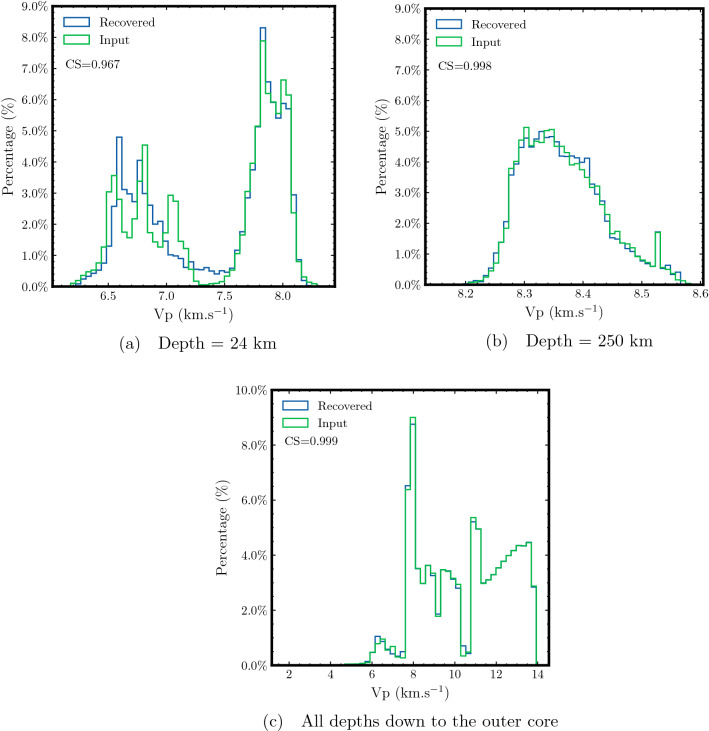
Comparison of velocity distributions between the target and recovered velocity models for the single point-source case. Distribution for the target (green) and the recovered (blue) velocities at a depth of 24 km (**a**) show a relatively good match for continental and oceanic parts of the lithosphere but the boundary zone between the two is smeared in the recovered velocity as the PINN model is unable to capture sharp velocity variations at this depth. However, the comparison at a depth of 250 km (**b**) shows striking similarity as the velocities at this depth have smoother variations. The comparison considering all the depth values (**c**) in the GLAD-M25 model show near-perfect recovery of the velocity model, highlighting the accuracy of traveltime predictions. The cosine similarity (CS) index is used to quantify the similarity between the distributions.

Next, we compare the velocity distribution between the target and recovered velocity models in Fig. 4. First, we compare the histogram of velocity values at a depth of 24 km in Fig. 4a. We observe a good match between the target and predicted velocities, particularly for higher velocity values corresponding to the continental part of the lithosphere. However, we observe a mismatch in velocity histograms for values corresponding to the boundary zone between the continental and oceanic lithosphere (velocities from 7 to 7.5 km/s). The recovered velocity is smeared in the region as it is unable to capture the rapid variations. On the contrary, in Fig. 4b we observe an excellent match between the target and predicted velocity histograms at a depth of 250 km. Finally, to have an idea of the overall accuracy across the entire model, Fig. 4c compares the velocity histograms for all depths in the GLAD-M25 model indicating a striking match between the target and recovered velocities. We confirm these observations through the cosine similarity (CS) metric. This metric quantifies an inner product between two normalized histograms. In other words, it quantifies the similarity distance as a function of the cosine angle^46^. The CS value of 0.999 for the entire model indicates overall high accuracy of traveltime predictions (CS value of 1 indicates identical distributions).

**Figure 5:**
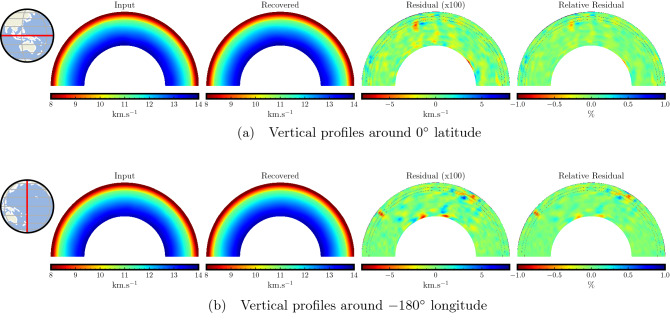
Accuracy analysis using vertical slices from the target and recovered velocity models and their difference for the single point-source case. A comparison of vertical profiles between the target and the recovered velocity models taken around the latitude of 0$^\circ$ (**a**) and the longitude of − 180$^\circ$ (**b**) show similar macro features. The velocity difference (blown up by 100 times) shows that the residuals are negligible and lie mostly between an absolute value of 0.02–0.05 km/s. The relative velocity residual confirms the agreement between the target and recovered velocity models.

We further analyze the accuracy of predicted traveltimes by comparing vertical slices of the two velocities taken around a latitude of 0$^\circ$ and a longitude of − 180$^\circ$, as shown in Fig. 5. We observe that the vertical slices show similar velocity features between the target and the recovered velocity models. Moreover, by looking at the residual values (blown up by 100 times) and the relative residual values, we observe negligible differences across depths, again an indication of the overall high accuracy of our traveltime predictions. We notice that while most of the residual values are near zero, the dominant absolute errors are merely around 0.02–0.05 km/s.

**Figure 6:**
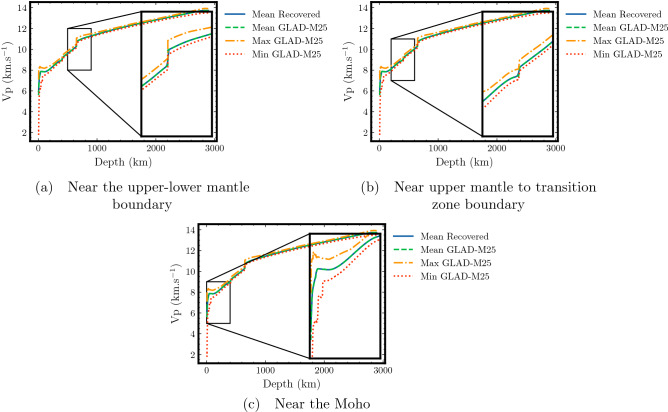
Comparison of 1-D velocity profiles at different seismic velocity discontinuities. The comparison between the mean GLAD-M25 and the recovered velocity models obtained by averaging all velocity values at each depth shows a close match. Zoomed-in views are presented to highlight the match at the three major seismic discontinuities. The minimum and maximum velocities for the GLAD-M25 velocity model at each depth are also shown.

Finally, we investigate the accuracy of the recovered velocity model by comparing 1-D velocity profiles. Figure 6 shows 1-D velocities corresponding to the average taken at each depth for the recovered and the GLAD-M25 velocity models. In addition, we show the minimum and maximum velocity values from the GLAD-M25 velocity model at each depth. Figure 6 is used to highlight velocity comparison at the three main seismic velocity discontinuities. The first discontinuity is along the crust-mantle boundary, the Moho discontinuity^47^, highlighted in Fig. 6a. The second discontinuity corresponds to the upper mantle and transition zone boundary, which is highlighted in Fig. 6b. The third discontinuity corresponds to the upper-lower mantle boundary, the 660 km discontinuity^48^, and highlighted in Fig. 6c. At all the three discontinuities, we observe a close match between the mean GLAD-M25 and the mean recovered velocities. Even for the largest discontinuity near the lithosphere, the average recovered velocity (solid blue line) matches the average target velocity values (dashed green line) fairly well (see Fig. 6a). This comparison highlights the fact that the NN is able to learn the underlying function and can produce accurate traveltimes, which were used to obtain the recovered velocity model. These discontinuities represent the main challenge for the algorithm as they are often difficult to capture using NNs due to their spectral bias^45^.

### USArray traveltime modeling

Having analyzed the accuracy of global seismic traveltime computation using PINNs for a single earthquake, we now turn towards a more realistic setting. We train the PINN model to predict traveltimes between any earthquake location in the global mantle Earth model to all the 2234 USArray stations. To speed up the training process, we initialize the neural network parameters using values from the previous training and use fine-tuning to update the weights. For efficient training, we use reciprocity between seismic sources and receivers and consider USArray stations as point-sources and randomly select points within the Earth model as receivers. This minor detail significantly speeds up the training process without affecting the training outcome. To keep the training time and memory requirements tractable, we use the same total number of training points as in the case of a single point-source. This results in a decreased coverage of the computational domain per USArray station as the total number of training points is distributed across the USArray stations. Once the training is complete, we analyze the accuracy of traveltime computation by considering each individual USArray station and predicting traveltimes from every point of the discretized GLAD-M25 model to the considered station. Specifically, the horizontal (longitude and latitude) grid spacing is 0.5$^\circ$ while the vertical discretization is performed such that the lithosphere is more finely sampled ($\approx$1 km) compared to the lower mantle layer ($\approx$16 km). These traveltimes are then used to compute the recovered velocity as before and compared with the target GLAD-M25 P-wave velocity model. We present the analysis considering two representative USArray stations for analyzing each depth presented here.

**Figure 7:**
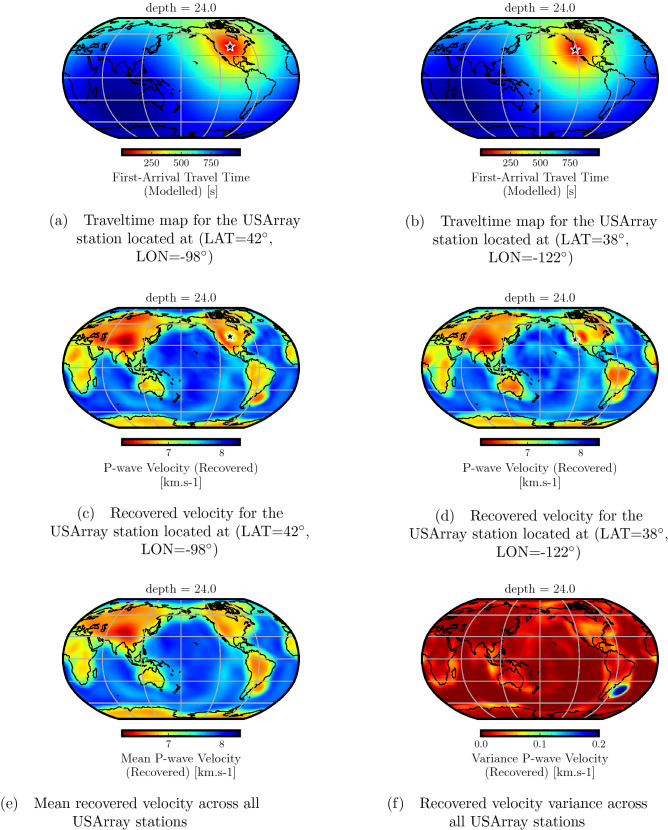
Analysis of recovered velocity for the USArray traveltime modeling case using a depth of 24 km. Traveltime maps for two representative USArray stations indicated by black stars (**a**,**b**) and their corresponding recovered velocity models (**c**,**d**) are shown. By comparing with the target velocity model at this depth (Fig. 2a), we observe similar velocity trends. Differences can be observed at boundary zone between the oceanic and the continental lithosphere which is due to the limited ability of the PINN model to capture sharp velocity variations in the lithosphere. The mean recovered velocity (**e**), taken by averaging over all USArray stations, shows the overall high accuracy of traveltime prediction. The recovered velocity variance (**f**) across all USArray stations shows small differences between the different recovered velocity models.

Figure 7a,b plot the predicted traveltime maps for the two considered USArray stations, while Fig. 7c,d show the recovered velocity for each station at a depth of 24 km, computed using these traveltime maps. While the two velocity models are largely similar, there are observable differences that are attributable to different ray coverage for each individual USArray station during the training process. By comparing these two recovered velocities with the target in Fig. 2a, we observe differences pre-dominantly at the boundary between the oceanic and continental lithosphere. This is understandable, as stated earlier, due to the sharp variation in velocity around this region. In Fig. 7e, we show the mean recovered P-wave velocity averaged over all recovered velocities for individual USArray stations indicating a similar trend. The variance of these recovered velocities is shown in Fig. 7f, indicating minor differences between the recovered velocities for each USArray station.

**Figure 8:**
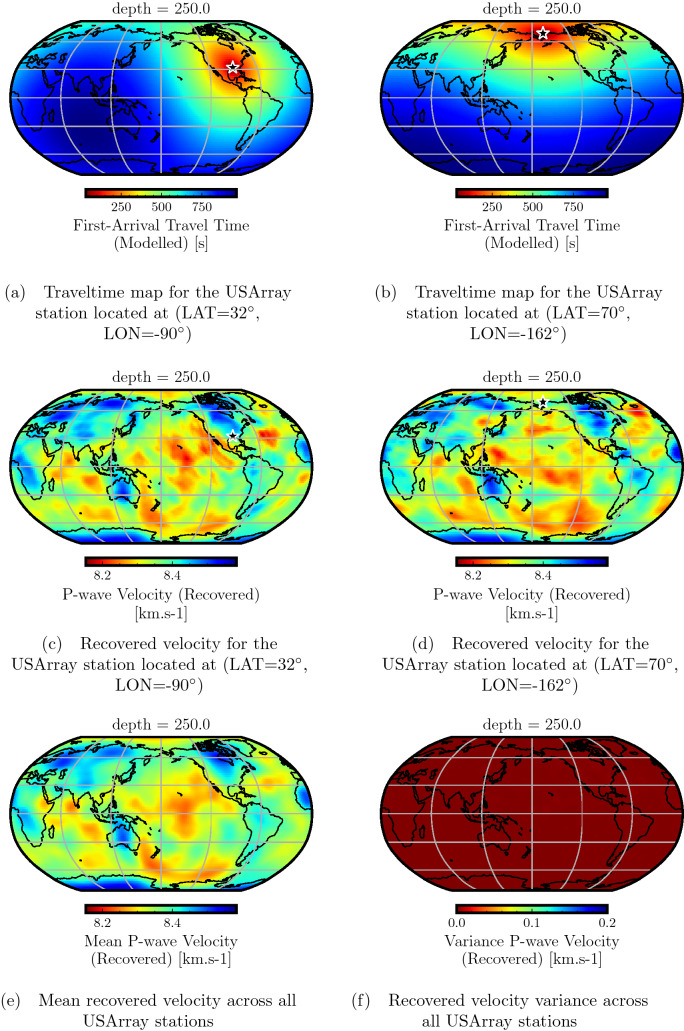
Analysis of recovered velocity for the USArray traveltime modeling case using a depth of 250 km. Traveltime maps for two representative USArray stations indicated by black stars (**a**,**c**) and their corresponding recovered velocity models (**b**,**d**) are shown. By comparing with the target velocity model at this depth (Fig. 3a), we observe similar velocity trends. Only minor differences are observed that are attributable to different ray coverage for each individual USArray station. The mean recovered velocity (**e**), taken by averaging over all USArray stations, shows the overall high accuracy of traveltime prediction. The recovered velocity variance (**f**) across all USArray stations shows negligible differences between the different recovered velocity models.

In Fig. 8, we perform a similar analysis for velocities at a depth of 250 km by considering two different USArray stations indicated using black stars. The traveltime maps corresponding to the two stations are shown in Fig.  8a,b. The recovered velocities using these traveltime maps are shown in Fig. 8c,d. Both the recovered velocities show similar trends with minor differences. The mean recovered velocity, obtained by averaging over all the USArray stations, is shown in Fig. 8e. It bears a close resemblance to the target velocity at this depth (see Fig. 3a), indicating accurate traveltime prediction for the entire USArray model from source points at this depth. Moreover, Fig. 8f shows the variance between the recovered velocities at different USArray stations, indicating minor differences between them.

**Figure 9:**
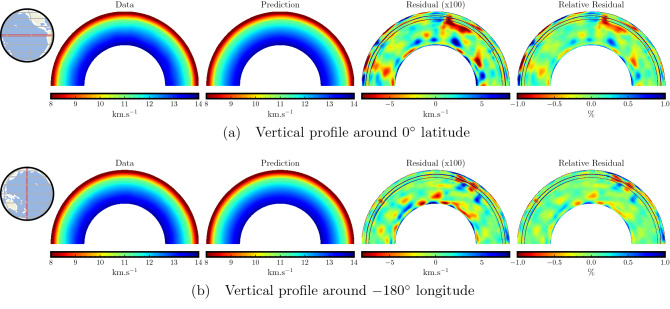
Accuracy analysis using vertical slices from the target and mean recovered velocity models and their difference for the USArray modeling test. A comparison of vertical profiles between the target and the mean recovered velocity models taken around a latitude of 0$^\circ$ (**a**) and a longitude of − 180$^\circ$ (**b**) show similar macro features. The velocity difference (blown up by 100 times) shows that the residuals are small and lie mostly below an absolute value of 0.08 km/s. The relative velocity residual confirms the agreement of the mean recovered velocity model with the target, highlighting the high accuracy of traveltime computation.

In Fig. 9, we compare the vertical slices around the latitude of 0$^\circ$ and the longitude of − 180$^\circ$. We again observe similar velocity macro-trends between the mean recovered velocity and the target velocity. The residual plots are blown up 100 times to highlight the differences. While the recovery is quite accurate for most parts, the error in the recovered velocity ranges merely between − 0.08 and 0.08 km/s. The relative residual plot confirms that the differences between the recovered and the target velocities are negligible, indicating accurate traveltime prediction for the entire USArray stations. Nevertheless, we notice that the accuracy, in this case, is slightly worse than the single-point source test (Fig. 5). This is because we use the same number of training points, but here, they are distributed over the entire USArray stations instead of focusing on a single point, resulting in slight degradation of accuracy.

### Global mantle traveltime modeling

We now analyze the extrapolation capability of our PINN model trained on the USArray to estimate traveltimes for stations beyond the USArray coverage. We consider three large magnitude earthquakes from different regions of the globe and estimate traveltimes from these earthquakes to hypothetical receivers lines covering part the USArray and extending beyond it. Furthermore, we also compare these traveltimes with observed traveltime values picked at seismic stations that lie on the considered hypothetical line of receivers. The coinciding seismic stations are part of the USArray and the International Seismological Centre (ISC) array.

**Figure 10:**
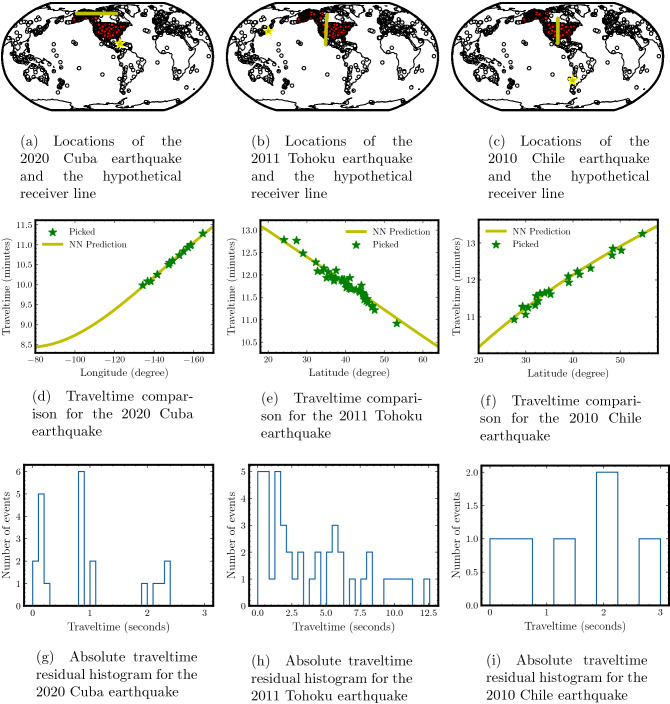
Analysis of our PINN model’s extrapolation ability beyond the training domain. Three large earthquakes from regions around the globe are chosen to study the generalization ability of our neural network trained for the USArray stations. Traveltimes are obtained using the trained neural network from these earthquake sources to hypothetical receiver lines shown in (**a**–**c**) for each case (**d**–**f**). Despite the fact that the hypothetical receiver lines extend beyond the area covered by the USArray, both traveltimes match pretty well. Also shown are the observed traveltimes picked at seismic stations lying on the hypothetical receiver line (green). Absolute traveltime residual histograms (**g**–**i**) highlight the overall accuracy of the neural network traveltime prediction despite using it to extrapolate beyond the training domain.

First, we consider the M$_w$ 7.7 earthquake that occurred on January 28, 2020 between Cuba and Jamaica. We estimate traveltimes from this earthquake to a dense line of receivers at a latitude of 60$^\circ$. The earthquake location and the receiver line are shown in yellow in Fig. 10a. Figure 10d compares the traveltimes obtained using the trained PINN model (solid yellow line) and those observed at the seismic stations (green stars) lying on this hypothetical line of receivers. The stations are located within a radius of 1$^\circ$ within the hypothetical line. We observe a close match between neural network predicted traveltimes and the observed earthquake events. Figure 10g plots the distribution of absolute traveltime residuals predicted by the trained neural network. The majority of the absolute erros are within 1 second. It is also worth mentioning that we do not perform any outliers removal to ensure the credibility of the residuals^49,50^.

Next, we perform a similar analysis for the M$_w$ 9.1 Tohoku earthquake that occurred on March 11, 2011 on the east coast of the Tohoku region in Japan. We estimate traveltimes from this earthquake to a dense line of receivers at a longitude of − 112$^\circ$. The earthquake location and the receiver line are shown in yellow in Fig. 10b. We also increase the radius to 2$^\circ$ within the hypothetical line to select the stations. The neural network predicted traveltimes are shown in Fig. 10e. We also plot traveltimes observed at seismic stations that lie on the considered hypothetical line of receivers. Compared to the previous case, the study is performed on the data recorded using the ISC array as opposed to the USArray (training data). Thus, with this setup we want to infer the generalization of the computed traveltime. Despite a slight reduction in the absolute residual values, as depicted in Fig. 10h, the trend clearly indicates that our method is capable of providing accurate predictions for traveltimes.

Finally, we analyze the extrapolation ability of the trained PINN model using the recording from the ISC array for the M$_w$ 8.8 Chile earthquake in the southern hemisphere, that occurred on February 27, 2010 off the coast of central Chile. We estimate traveltimes from this earthquake to a dense line of receivers at a longitude of − 100$^\circ$, as shown in Fig. 10c. The neural network predicted traveltimes are shown in Fig. 10f along with those observed at the actual seismic stations, with the same radius selection of 2$^\circ$, lying within this line of receivers. Again, we observe good match between the predicted traveltimes and the observed values.

These examples show that our trained PINN model is capable of providing accurate traveltimes from any earthquake location to not only the area covered by the USArray but even beyond. Moreover, the traveltime prediction using the trained PINN model (performed on a CPU for fair comparison) is an order of magnitude faster than computing them using a 1-D velocity model calculated using a 1-D ray tracing *Python* library, *ObsPy*^51^, as well as a 3-D ray tracing method^52^. For a single source-receiver pair, the computational time required by our trained method is only 0.05 milliseconds. In comparison, 1-D and 3-D ray tracing methods require 32.84 seconds and 61.40 seconds, respectively, illustrating the efficiency of our approach. While these ray-tracing codes may be further optimized to improve their efficiency, the idea here is to compare our method with open-source tools that are readily available and routinely used by seismologists.

## Discussion

We have developed a neural network based method to rapidly estimate global seismic traveltimes using advances in the field of physics-informed machine learning. By minimizing a loss function constructed from the eikonal equation (and its boundary conditions), a neural network is trained to compute the traveltime between any two points in a global mantle model. Once the model is trained, it can handle any number of source-receiver pairs efficiently ($\sim$ 47 μs per pair). This marks a significant stride forward in computational seismology as conventional finite-difference based eikonal solvers, or even ray tracing methods, are unable to handle millions of receivers made available by emerging acquisition technologies such as distributed acoustic sensing. Through extensive numerical tests, we show that our method is capable of producing largely accurate traveltimes in a computationally efficient manner and scales independently of the number of receivers. Our trained neural network can be used as an efficient forward modeling engine for speeding up seismic inversion algorithms at the global scale, potentially leading us into a new era of seismological discoveries.

The highlighted advantages are made possible thanks to the ability of neural networks to approximate any continuous, bounded function. Once trained, a neural network can produce a continuous function output with respect to its inputs. Therefore, we can produce seismic traveltime maps that are mesh-independent. Moreover, these traveltimes can be obtained on the fly and, unlike conventional methods, there is no need to store traveltime look up tables. The memory requirement, in this case, is dictated by the neural network architecture as only the network parameters need to be stored. Furthermore, the trained neural network may also be seen as an efficient storage mechanism of the global seismic velocity model as it learns the velocity representation during the training process. So we can extract the traveltime or velocity information from the network at any point. Thus, instead of using conventional interpolation techniques to overcome the first-order traveltime inaccuracy^15^, we utilize the neural network’s non-linear interpolation ability, optimized to fit the eikonal PDE. Another advantage of our method is that the traveltime solution is guaranteed to be differentiable with respect to the source or receiver locations. This allows the method to be used for a variety of seismological applications, such as earthquake source localization as it requires computation of gradients of an objective function with respect to source locations, which are readily available. Moreover, our approach enables a straightforward computation of high-order derivatives of traveltimes, which can be valuable in computing raypaths and amplitudes, and it also facilitates an efficient computation of seismograms for doubly-scattered waves. We also show the generalization capabilities of our approach by predicting accurate traveltimes beyond the region covered by the training process.

Our implementation is made efficient thanks to the state-of-the-art GPU hardware and modern deep learning libraries like PyTorch, allowing rapid calculation of gradients through automatic differentiation. The training of our neural network takes about 52 min per epoch on a single NVIDIA GeForce GTX TITAN GPU. However, once trained, the neural network can produce traveltimes rapidly through a single evaluation of the network, making the approach attractive particularly when a large number of sources and receivers are involved. Our approach is massively parallel and best suited for GPU hardware, taking only a fraction of a second to compute traveltimes at the global scale.

Apart from the mentioned training challenges, our method is driven by the eikonal formulation of traveltime, which is based on a high-frequency asymptotic approximation of the wave equation. Its numerical solution often admits the viscosity solution, which tracks the first-arrivals. Based on our tests, these traveltimes match well with picked first-arrival earthquake traveltime data often used for locating earthquakes and performing P-phase arrival tomography. Nevertheless, it is worth highlighting that the ability to predict first-arrival traveltimes can be a serious limitation for traveltime tomography in the upper mantle and transition zones where the use of multi arrivals are quite important^53,54^. While the issue of computing multiple arrival traveltimes using our approach remains an outstanding challenge, the concept of raylets^55^ can be used as first step towards addressing it. Moreover, the approximated traveltime gradients is assumed to be well behaved which may be inaccurate in the face of severe discontinuities (e.g., caustics). The other important consideration is that here we only model the P-phase; the proposed workflow can be used to obtain the S-phase by using the shear velocity in Eq. (1).

Going forward, the accuracy of our results can be further enhanced with the increasing computational capabilities of GPUs and our increased understanding of the training dynamics for physics-informed neural networks (PINNs). Moreover, the traveltime computation can be made more accurate by considering more realistic physics of the Earth model, including the anisotropic and attenuation effects of seismic wave propagation. The transfer learning approach can be applied to compute traveltimes for a different velocity model (e.g., an updated version of the GLAD-M25 model). However, it is not obvious that the same network will be able to capture the higher wavenumber components of a different velocity model as that is determined by the expressivity of the current architecture. Neuron splitting offers an opportunity to expand the size of the network while utilizing the learned features^56^. It might provide a path for capturing higher resolution information introduced into the updated velocity model. In addition, the sensitivity matrix can be evaluated from the gradient of the additive traveltime field, which is obtained from the source and receiver pairs at all points in the domain^38^. The flexibility of the PINN framework allows embedding additional physics into the workflow by merely updating the loss function corresponding to the correct form of the eikonal equation.

## Methods

We train a neural network model to learn a traveltime function using the global Earth velocity model. Once trained, the neural network can be used to instantly obtain the traveltime between any two points in a 3-D Earth model. Furthermore, the trained neural network can be used as an efficient storage mechanism for the global Earth velocity model that can be queried on the fly for seismological applications. Below, we summarize the key elements for achieving these objectives.

### The eikonal equation

The eikonal equation for an isotropic medium can be written as

1 $$\begin{aligned} |\nabla T({{\textbf {x}}})|^2 = \frac{1}{V^2({{\textbf {x}}})}, \end{aligned}$$

where *T* denotes the traveltime field and *V* denotes the medium velocity, both as a function of the position vector ${{\textbf {x}}}$. The eikonal equation simply states that the magnitude of the gradient of the arrival time surface is inversely proportional to the speed of the wavefront. The traveltime field is also constrained by location of the source, $x_s$, in which we assume that $T(x_s)$ = 0.

Instead of solving the original form of the eikonal equation, we decompose the traveltime field into two multiplicative functions and obtain the factored form of the eikonal equation^57^:

2 $$\begin{aligned} T({{\textbf {x}}}) = \tau ({{\textbf {x}}})T_0({{\textbf {x}}}), \end{aligned}$$

where a scalar $\tau$ is introduced to map the background traveltime $T_0$ to the actual traveltime *T*. We choose the background traveltime to be simply the distance between two points (source ${{\textbf {x}}}_S$ and location ${{\textbf {x}}}$ in the domain of interest) divided by a background constant velocity $V_0$. Hence, Eq. (2) can be rewritten as

3 $$\begin{aligned} T({{\textbf {x}}}_S, {{\textbf {x}}}) = \tau ({{\textbf {x}}}_S, {{\textbf {x}}})\frac{\Vert {{\textbf {x}}} - {{\textbf {x}}}_S\Vert }{V_0}, \end{aligned}$$

resulting in $\tau ({{\textbf {x}}}_S, {{\textbf {x}}})$ as the unknown to be solved for. The factored form allows us to absorb the point-source singularity in the analytical background traveltime *T* rendering the unknown function $\tau$ well behaved and smooth in the neighborhood of the point-source. This allows a neural network to approximate the function $\tau ({{\textbf {x}}}_S, {{\textbf {x}}})$ faster than $T({{\textbf {x}}}_S, {{\textbf {x}}})$ due to the well-known bias of neural network learning towards smooth functions^45^.

### Physics-informed neural network optimization

Thanks to the universal approximation theorem^42^, we can approximate the functional solution of a PDE using a neural network. The traveltime factor $\tau$ is approximated via a neural network functional *f*, which is parameterized by its weights and biases, $\varvec{\Theta }$, and has inputs as the source and receiver coordinate vectors ${\textbf {x}}_S$ and ${{\textbf {x}}}$, respectively. This can be formally expressed as:

4 $$\begin{aligned} \tau ({{\textbf {x}}}_S, {{\textbf {x}}})\approx f({{\textbf {x}}}_S, {{\textbf {x}}}; \Theta ). \end{aligned}$$

The gradient of $\tau ({{\textbf {x}}}_S, {{\textbf {x}}})$ w.r.t. ${{\textbf {x}}}$ can be evaluated directly using the chain rule and implemented through automatic differentiation^43^.

Once the traveltime field for a given point-source is known, the eikonal equation can be used to explicitly calculate the corresponding velocity. If we plug Eq. (2) into Eq. (1), for a particular source, we end up with

5 $$\begin{aligned} \begin{aligned} V({{\textbf {x}}})&=\left[ \left( T_{0}({{\textbf {x}}})\nabla _{{\textbf {x}}}\tau ({{\textbf {x}}}) + \nabla _{{{\textbf {x}}}}T_0({{\textbf {x}}})\tau ({\textbf {x}})\right) ^2\right] ^{-\frac{1}{2}}, \\&= \left[ T_{0}^{2}\left\| \nabla _{{{\textbf {x}}}} \tau ({\textbf {x}})\right\| ^{2}+ \frac{2 \tau ({{\textbf {x}}})\left( {{\textbf {x}}}-{\textbf {x}}_S\right) \cdot \nabla _{{{\textbf {x}}}} \tau ({\textbf {x}})}{V_0^2}+\frac{\tau ({{\textbf {x}}})^{2}}{V_0^2}\right] ^{-\frac{1}{2}}, \end{aligned} \end{aligned}$$

and by plugging in ${{\textbf {x}}}={{\textbf {x}}}_S$ in search for the boundary condition (values of $\tau ({{\textbf {x}}}_S)$), Eq. (5) yields

6 $$\begin{aligned} V({{\textbf {x}}}={{\textbf {x}}}_S)=\frac{V_0}{\tau ({{\textbf {x}}}_S)}. \end{aligned}$$

Therefore, using Eqs. (5) and (6), we construct a loss function to train our PINN model, which is given as

7 $$\begin{aligned} J =\frac{1}{N_{{{\textbf {x}}}_T}}\sum _{({{\textbf {x}}}) \in {{\textbf {x}}}_T} \left| \frac{{\hat{V}}({{\textbf {x}}})-V({{\textbf {x}}})}{V({{\textbf {x}}})}\right| ^2 + \left| \frac{\tau _{{{\textbf {x}}}_S} - \frac{V_0}{V({{\textbf {x}}}={\textbf {x}}_S,{{\textbf {x}}}_S)}}{\frac{V_0}{V({{\textbf {x}}}={{\textbf {x}}}_S,{\textbf {x}}_S)}}\right| ^2. \end{aligned}$$

The first term of the loss function *J* corresponds to the minimization of the relative difference between the given target velocity $V({{\textbf {x}}})$ and the recovered velocity ${\hat{V}}({\textbf {x}})$. The latter is obtained by plugging the approximated spatial gradient $\nabla \tau ({{\textbf {x}}}_S,{{\textbf {x}}})$ from the trained neural network into Eq. (5). The second term in Eq. (7) corresponds to the boundary condition. In other words, the neural network is encouraged to admit the true value of $\tau ({{\textbf {x}}}_S,{{\textbf {x}}})$ at the source, which can be easily obtained using Eq. (6). These two terms ensure that the governing eikonal equation is indeed satisfied (first term), and the necessary boundary condition (second term) is also honored during the minimization of the loss function. This optimization process is performed on a randomly chosen set of training points $N_{{\textbf {x}}_T}$.

### Network architecture and workflow

As shown in Fig. 1, we use a feed-forward neural network including residual blocks. The network takes an input of two three-dimensional vectors (a total of six inputs) that correspond to the source and receiver coordinates in a 3-D Earth model. These input vectors may correspond to any coordinate system, but for a non-Cartesian system, a pre-processing step is needed to ensure the input vector values range between − 1 and 1. This can be achieved, for example, by performing a geodetic (spherical) to geocentric (Cartesian) coordinate transformation and scaling with its largest absolute value. The network consists of fully-connected layers as the first and the last hidden layer with a sequence of 20 residual blocks in the middle. In all of these layers, 512 neurons are used. The output of the network is the scalar factor $\tau$, which maps the background traveltime $T_0$ to the actual traveltime field *T*. The backpropagation algorithm is then used to compute the gradient of $\tau$ w.r.t. the spatial coordinates ${{\textbf {x}}}$, which is required by the eikonal equation. We then train the neural network in a semi-supervised manner by incorporating the eikonal equation in the loss function *J*. The target velocity model at the receiver location $V(\textbf{x})$ is provided to compute the loss function.

Once the network is trained, we can compute traveltimes between chosen source and receiver coordinates $({{\textbf {x}}}_S,{{\textbf {x}}})$ through a single evaluation of the neural network and without the need for the global velocity model as it is embedded in the neural network parameters. This allows us also to access the global Earth velocity at any point (no interpolation between grid points needed) using Eq. (5). We will use this feature to compute the recovered velocity for validating the traveltime accuracy against the target (input) velocity.

### Implementation details

Below, we elaborate on the implementation details including the 3-D velocity model used, input pre-processing steps, and details of the training process.

#### Input velocity model

We use the compression wave velocity from the second generation of the 3-D global adjoint tomography model, GLAD-M25^44^. The model is built to account for realistic effects due to 3-D anelastic behavior of the Earth, topographic and bathymetric variability, as well as Earth’s ellipticity, self-gravitation, and rotation. Using this model allows us to compute more accurate traveltimes compared to the standard 1-D velocity models used (e.g., the *ek137* model from^58^) for simplicity.

The input coordinates for the neural network are sampled from the original points on the vertically-polarized P-wave velocity from the GLAD-M25 model. These points are sampled randomly from a coarser representation (four times larger than the initial sampling) of the GLAD-M25 model along the longitude and latitude dimensions. Hence, the total points for the training and validation process are 21, 627, 871. Out of these points, we allocate 90% for training and 10% for validation.

#### Projection and normalization

We perform a coordinate system projection and normalization to the input of the network to ensure stable training. A projection step transforms the input from a geodetic coordinate system ($\theta ,\phi ,r$) to a geocentric coordinate system (*X*, *Y*, *Z*). This step is introduced to make the eikonal formulation inline with the GLAD-M25 model, which is made on top of the SPECFEM3D GLOBE^59^ algorithm. This algorithm internally uses the Cartesian coordinate for the numerical integration of the spectral-element method. Thus, although the input velocity uses a more natural geodetic coordinate system, a projection step appropriately accommodates the Earth’s first-order dependency on it’s radius. Finally, a division by the the values of the average radius of the Earth (6371 km) is performed to the projection output. This completes the normalization step and warrants the inputs to the network to be in the range of $[-1, 1]$.

#### Training details

The neural network architecture details and hyper-parameter values are summarized in Table 1. We use skip connections for the regression problem in the form of residual blocks. The idea originated from the successful implementation of skip connections on image recognition problems^60^. Using the same idea, by introducing the skip connections, the model is expected to learn more complex features compared to only fully connected layers. Given a large number of training points, each epoch takes around 52.4 minutes on a single NVIDIA GeForce GTX TITAN GPU. However, once the neural network is trained, the inference process is noticeably faster than even a standard eikonal solver (e.g., the fast-marching method (FMM)^61^).

**Table 1:** Implementation details of the training process.

No.	Hyperparameter	Values/Type
1	Number of residual blocks	20
2	Number of neurons per layer	512
3	Activation function	ELU
4	Optimization algorithm	Adam
5	DL framework/wrapper	PyTorch
6	Trainable parameters	16,554,039
7	Number of points in GLAD-M25	89,016,102
8	Learning rate	$1e^{-6}$
9	Batch size	128
10	Number of training epochs	160
11	Number of training points	19,465,083
12	Weight initialization	Xavier normal

## Data Availability

The data used in this study are publicly available at http://ds.iris.edu/ds/products/emc-glad-m25/. All the source codes to reproduce the results in this study are accessible through GitHub at https://github.com/hatsyim/globenn.
